# Characterization of Squamosa-Promoter Binding Protein-Box Family Genes Reveals the Critical Role of *MsSPL20* in Alfalfa Flowering Time Regulation

**DOI:** 10.3389/fpls.2021.775690

**Published:** 2022-01-05

**Authors:** Lin Ma, Xiqiang Liu, Wenhui Liu, Hongyu Wen, Yongchao Zhang, Yongzhen Pang, Xuemin Wang

**Affiliations:** ^1^Institute of Animal Science, Chinese Academy of Agricultural Sciences, Beijing, China; ^2^College of Grassland Science and Technology, China Agricultural University, Beijing, China; ^3^Key Laboratory of Superior Forage Germplasm in the Qinghai-Tibetan Plateau, Qinghai Academy of Animal Science and Veterinary Medicine, Qinghai University, Xining, China

**Keywords:** alfalfa (*Medicago sativa* L.), expression patterns, *MsSPL20*, flowering time, *SPL* family genes

## Abstract

*SQUAMOSA Promoter-binding protein-Like* (*SPL*) genes affect a broad range of plant biological processes and show potential application in crop improvement by genetic modification. As the most widely planted forage crop in the world, biomass and abiotic stresses tolerance are important breeding targets for alfalfa (*Medicago sativa* L.). Nevertheless, the systematic analysis of *SPL* genes in alfalfa genome remains lacking. In the present study, we characterized 22 putative non-redundant *SPL* genes in alfalfa genome and uncovered the abundant structural variation among *MsSPL* genes. The phylogenetic analysis of plant SPL proteins separated them into 10 clades and clade J was an alfalfa-specific clade, suggesting *SPL* genes in alfalfa might have experienced gene duplication and functional differentiation within the genome. Meanwhile, 11 *MsSPL* genes with perfect matches to miRNA response elements (MREs) could be degraded by *miR156*, and the cleavage sites were gene specific. In addition, we investigated the temporal and spatial expression patterns of *MsSPL* genes and their expression patterns in response to multiple treatments, characterizing candidate *SPL* genes in alfalfa development and abiotic stress tolerant regulation. More importantly, overexpression of the alfalfa-specific *SPL* gene (*MsSPL20*) showed stable delayed flowering time, as well as increased biomass. Further studies indicated that *MsSPL20* delayed flowering time by regulating the expression of genes involved in floret development, including *HD3A*, *FTIP1*, *TEM1*, and *HST1*. Together, our findings provide valuable information for future research and utilization of *SPL* genes in alfalfa and elucidate a possibly alfalfa-specific flowering time regulation, thereby supplying candidate genes for alfalfa molecular-assisted breeding.

## Introduction

Over the past two decades, *SQUAMOSA Promoter-binding protein-Like* (*SPL*) genes, which encode a class of plant-specific transcription factors, have been shown to affect a broad range of plant biological processes (Chen et al., 2010). These processes include the timing of vegetative phase changes and floral induction, the rate of leaf initiation, shoot regeneration and branching, anthocyanin and trichome production, stress responses, carotenoid biosynthesis, and lateral root development (Gou et al., 2011; Jung et al., 2012; Cui et al., 2015; Zhang et al., 2015; Ioannidi et al., 2016; Preston et al., 2016). SPL proteins are characterized by the presence of a highly conserved SQUAMOSA-PROMOTER BINDING PROTEIN (SBP) domain which is consisted of approximately 78 amino acid residues containing a nuclear localization signal (NLS) motif and two Zn finger-like structural motifs (Yang et al., 2008). *SPL* genes regulate the transcription of downstream genes through the binding of the SBP domain to GTAC core motif, thereby participating in the regulation of plant growth and development (Birkenbihl et al., 2005). In addition, most *SPL* genes can be degraded by miRNAs and the miRNA Responsive Element (MRE) lie downstream of the conserved SBP domain (Gandikota et al., 2007; Molnar et al., 2007).

Since the cloning and investigation of the first two *SPL* genes, *AmSBP1* and *AmSBP2*, in *Antirrhinum majus* (Klein et al., 1996), *SPL* genes have been identified and characterized from various plant species, including *Arabidopsis*, rice, wheat, maize, cotton, soybean, etc. (Cardon et al., 1999; Xie et al., 2006; Zhang et al., 2014; Tripathi et al., 2017; Cai et al., 2018; Wei et al., 2018). The classification of the *SPL* family has been contentious, with six to nine major clades identified by various researchers through neighbor-joining phylogenetic analysis (Guo et al., 2008). Nevertheless, phylogenetic evidence supports the derivation of multiple *SPL* paralogs from the ancestral *SPL* genes following gene duplication and speciation, which have been maintained in the genome by positive Darwinian selection (Preston and Hileman, 2013; Zhong et al., 2019). These findings indicate the vital roles of *SPL* genes in plants, as positive Darwinian selection reflects adaptation to novel ecological conditions.

Recent studies have suggested that *SPL* genes participate in the regulation of multiple agronomic traits and demonstrated their potential application for crop genetic modification (Wang and Wang, 2015). The most elucidated *SPL* genes, *OsSPL14/IPA1* (*Ideal Plant Architecture1*), have been found to directly activate the expression of *OsTB1* (*Teosinte Branched1*) and *OsDEP1* (*Dense and Erect Panicle1*), which regulate rice tilling and panicle morphology, respectively (Wang J. et al., 2017). Moreover, *OsSPL14* acts on *OsD53* (*Dwarf53*) via a feedback regulatory mechanism to mediate strigolactone (SL)-regulated tiller development (Song et al., 2017). On the other hand, *SPL* genes play critical roles in the regulation of plant biotic and abiotic stress tolerance. *SPL9* has been found to balance reproduction and survival by directly regulating the biosynthesis of anthocyanin through *PAP1* (*production of anthocyanin pigments1*) and *DFR* (*dihydroflavonol-4-reductase*) (Cui et al., 2015). Over-expressing of *BpSPL9* in birch (*Betula platyphylla* Suk.) improves the scavenging of ROS under abiotic stress, thus indicating the strong contribution of this gene to salt and drought resistance (Ning et al., 2017).

Alfalfa (*Medicago sativa* L.) is the most important, widely grown forage plant in the world because of its high biomass, notable adaptability, exceptional nutritive value, and remarkable biological nitrogen fixation capacity (Russelle et al., 2007; Gou et al., 2018). Given the critical roles of *SPL* genes in plants, several *SPL* genes in alfalfa have been reported to participate in the regulation of multiple developmental processes and abiotic stress tolerance (Gao et al., 2018; Gou et al., 2018; Feyissa et al., 2019, 2021; Lorenzo et al., 2019). For instance, transgenic alfalfa silencing *MsSPL13* displays more lateral branches and delayed flowering time, and the shoot branching genes were significantly down-regulated in *SPL13* RNAi plants (Gao et al., 2018). In shaded alfalfa plants, the expression level of *MsSPL3* is significantly down-regulated; and overexpression of *MsSPL3* in *Arabidopsis* resulted in an early flowering phenotype (Lorenzo et al., 2019). Research on an enhanced branching mutant of *Medicago truncatula* reveals that the loss of function of *spl8* increases biomass, while the over-expression of *SPL8* inhibits branching by suppressing axillary bud formation; the latter is also true for *MsSPL8* in alfalfa (Gou et al., 2018). Despite all of factors, a systematic analysis of *SPL* genes in alfalfa genome still remains lacking. As an important trait in alfalfa, the timing of flowering (TOF) guides the determination of harvesting time since farmers often cut alfalfa at the early bloom stage, which helps to balance forage quality and biomass (Adhikari et al., 2019). Recently, information on the genome of autotetraploid alfalfa has been made public, allowing us to perform a detailed systematic analysis of *SPL* genes in alfalfa and helping us to understand the genetic and genomic basis of alfalfa flowering time regulation (Chen et al., 2020; Shen et al., 2020).

In the present study, we characterized 22 putative non-redundant *SPL* genes in alfalfa. We uncovered abundant structural variation among the 22 *MsSPL* genes, and a phylogenetic analysis of plant SPL proteins separated them into 10 clades with an alfalfa-specific clade (J). Moreover, we found that 11 *MsSPL* genes with perfect matches to MRE could be degraded by miR156, and the cleavage sites were gene specific. We also investigated the temporal and spatial expression patterns of *MsSPL* genes. In addition, the expression patterns of *MsSPL* genes under normal growth conditions and in response to multiple treatments were also measured. More importantly, transgenic alfalfa over-expressing *MsSPL20* (a alfalfa-specific *SPL* gene) showed a stable delayed flowering time phenotype, as well as increased biomass. Further RNA-seq analysis demonstrated the possible molecular mechanism of *MsSPL20* in alfalfa flowering time regulation. The detailed results presented here provide valuable information for future research and utilization of *SPL* genes in alfalfa, and contribute to elucidating the genetic basis of flowering time regulation in alfalfa, thereby supplying candidate genes for alfalfa molecular-assisted breeding.

## Materials and Methods

### Plant Materials and Treatments

Alfalfa seeds (*Medicago sativa* L. cultivar Zhongmu No. 1) were surface sterilized with 70% (v/v) ethanol for 5 min, swilled with ddH_2_O several times, and then sown in pots with soil. The seedlings were grown at 25°C (14 h/10 h of light/dark) in a greenhouse and watered every 5 days. The investigated tissues, including root, stem, head, leaf, flower, and seedpod, in various growing stages were sampled for tissue-specific expression analysis.

The seeds of Zhongmu No. 1 were surface sterilized and germinated on filter paper for 7 days; then, seedlings with a primary root length of 1.5 cm were transferred to a hydroponics system with 1/2 MS nutrient solution and grown in a greenhouse until the third compound leaf unfolded. The seedlings were, respectively, watered with 200 mM NaCl, 18% (w/v) PEG 6000, and exposed to 4°C for simulating saline, drought, and cold treatments. For plant hormone treatments, GA3, GR24, IAA, and ABA at 0.1 mM concentration were added to the culture solution. The seedlings in each treatment were collected at 0, 2, 12, and 24 h. All samples were immediately frozen in liquid nitrogen and stored at –80°C for subsequent RNA extraction.

### RNA Extraction and Gene Expression Analyses

Total RNA was extracted using an Eastep™ Super Total RNA Extraction Kit (Promega; code LS1040) and the first-strand cDNA synthesis was performed with TransScript One-Step gDNA Removal and cDNA Synthesis SuperMix (TransGen; code AT311) according to the manufacturer’s instructions. Quantitative real-time PCR was conducted on an ABI QuantStuio 7 Flex RT-PCR instrument with SYBR Premix Ex Taq (Tokoya; code RR820A) according to the manufactures’ instructions. The relative expression levels of target genes were calculated using the –2^△△CT^ method. The specific primers used for qRT-PCR are listed in Supplementary Table 1.

### Identification and Gene Structure Analyses of *SQUAMOSA Promoter-Binding Protein-Like* Family Members in Alfalfa

Two strategies were used to search for the members of the *SPL* family genes in XinJiangDaYe genome.^1^ First, the SBP domain (PF03110) protein sequence was used as query sequence to carry out BLASTP search with an *E*-value cutoff of 1E^––4^. Additionally, *SPL* genes from other species were used as query sequences to perform BLASTN searches to find out *SPL* genes in alfalfa. The redundant sequences were subsequently removed from the obtained sequences. Finally, domain analysis programs in SMART^2^ were applied to confirm that if the obtained sequences were likely to be SPL proteins. The molecular weight (MW) and isoelectric point (PI) of each protein were calculated using ExPASy.^3^ The exon/intron structure of *SPL* genes was determined based on the alignment of the open reading frame (ORF) sequences with their corresponding genomic sequences, and the corresponding structure diagrams were acquired by using the Gene Structure Server (GSDS 2.0).^4^

### Chromosomal Distribution and Collinearity Analyses

The schematic diagram of chromosomal distributions of *MsSPL* genes were drawn by using MG2C software, based on their location information and chromosomal length of alfalfa.^5^ Collinearity analysis of the *SPL* genes in alfalfa genome was performed by using multiple collinear scanning toolkits (MCScan X) with an *E*-value set to 10^–5^ (Wang Y. P. et al., 2012).

### Phylogenetic and Conserved Motif Analyses of *SQUAMOSA Promoter-Binding Protein-Like* Proteins

A total of 57 SPL proteins of related plants, including 19 from rice (Xie et al., 2006), 16 from *Arabidopsis* (Cardon et al., 1999), and 22 from alfalfa, were selected for phylogenetic analysis. An un-rooted phylogenetic tree was constructed by using MEGA 7.0^6^ followed by multiple sequence alignments via the neighbor-joining method (NJ), and the bootstrap analysis was conducted using 1,000 replicates and gaps/missing data were treated by complete deletion. MEME Suite Version 4.12.0^7^ was used to detect the conserved domains of SPL proteins with the following parameters: width of each motif was 20–200 amino acid residues; the maximum number of motifs was 3; and other parameters were set to default values.

### Validation of miRNA Cleavage Site by RNA Ligase-Mediated 5′RACE Assay

A previously reported RNA ligase-mediated 5′RACE (RLM-5′-RACE) assay was employed to validate miRNA cleavage sites by using a modified RLM-RACE kit (Invitrogen; code D315) in alfalfa (Li et al., 2015). Briefly, approximately 2 μg of total RNA was used for the ligation of an RNA oligo adaptor without calf intestinal phosphatase treatment. For the first round of PCR, the 5′RACE outer primer together with gene-specific outer primer was used, and a nested PCR amplification was then carried out using the 5′RACE inner primer together with gene-specific inner primer. The obtained PCR products were then cloned into vector for sequencing.

### Subcellular Localization and *Trans-*Activation Activity Assay

The full-length coding sequence of *MsSPL20* without the stop codon was fused upstream of the green fluorescent protein (GFP) under the control of the CaMV 35S promoter to generate *pCAMBIA1302:MsSPL20-GFP*. The recombinant plasmid was confirmed by sequencing and transferred into *Agrobacterium* strain *GV3101* using the freezing/heat-shock method. The sub-cellular localization of the fusion protein was investigated with a tobacco transient expression system (Liu et al., 2017). For *trans*-activation activity assays, full-length *MsSPL20* was cloned into the pGBKT7 vector to construct BD-MsSPL20. The detailed assay was performed according to a previous report (Ma et al., 2019).

### Transformation and Transcriptome Assays

For transformation assay, the coding sequence of *MsSPL20* was obtained through PCR amplification using MsSPL20-F and MsSPL20-R and inserted into the modified pBI121 vector (GUS deletion) through seamless cloning (Aidlai, Lot: CV1901). The verified construct *pBI121:MsSPL20* was transferred into *Agrobacterium* strain *GV3101* using the freezing/heat-shock method. Transgenic alfalfa plants were obtained by *Agrobacterium*-mediated transformation as previously reported (Wang X. M. et al., 2017).

For transcriptome assay, the selected transgenic alfalfa and control plants were propagated using shoot cuttings. Total RNA samples from three biological replicated of OE and control plants were isolated from the mixed leaves and heads tissues which were cultured as consistent as possible. RNA-seq and the following data analysis were completed by Genedenovo Biotechnology Co., Ltd. (Guangzhou, China).

## Results

### Genome-Wide Identification and Bioinformatic Analyses of *MsSPL* Genes

**FIGURE 1:**
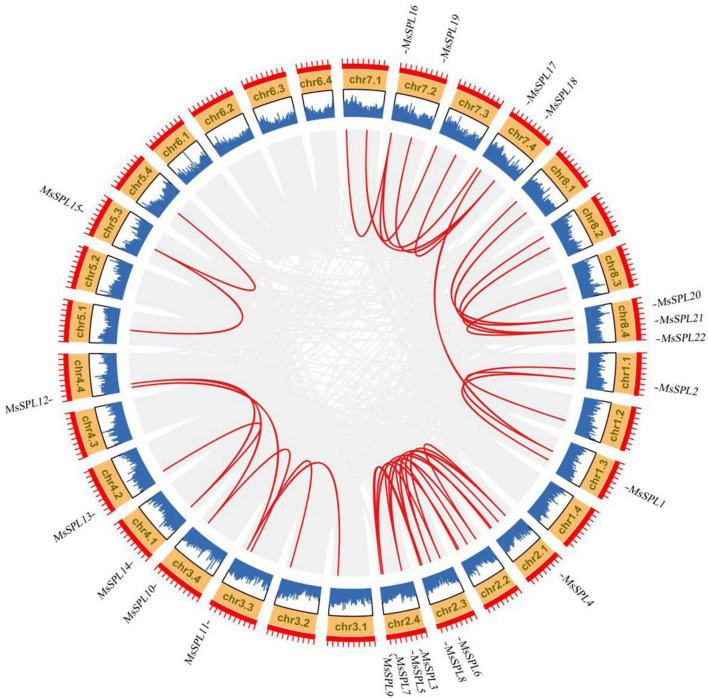
Chromosomal distribution of *MsSPL* genes with the alfalfa genome. Gray lines in the background indicate the collinear blocks in alfalfa genome. Red lines highlight the synthetic relationship of *MsSPL* genes.

Through BLAST searches and domain conformation analyses, we identified 84 putative *SPL* gene sequences in XinJiangDaYe genome (see text footnote 1). After confirming the SBP domain in SMART, 72 of them contained SBP domain and distributed in alfalfa chromosomes except Chr1.4, 4.3, 5.2, 6.1, 6.2, 6.3, and 6.4 (Supplementary Figure 1). We subsequently clustered these 72 putative *SPL* genes and obtained 22 non-redundant *MsSPL* genes, including their CDSs and genomic sequences in alfalfa (Supplementary Figure 2). These 22 *MsSPL* genes were randomly distributed on 7 chromosomal groups and were designated as *MsSPL1*–*MsSPL22* on the basis of their chromosomal locations (Figure 1). The molecular weight of these 22 MsSPL proteins ranged from 16.58 kD (MsSPL8) to 130.58 kD (MsSPL7), and their isoelectric points varied from 5.66 (MsSPL3) to 9.23 (MsSPL17) (Supplementary Table 2).

Exon/intron and conserved motif analyses showed that *MsSPL* genes varied in gene structure and motifs. Ten *MsSPL* genes (*MsSPL1*, *5*, *11*, *12*, *15*, *17*–*20*, and *22*) had the classical *SPL* gene structure, namely, three exons and two introns, while the remaining genes contained 2 (*MsSPL3*, *4*, *8*, *9* and *21*), 4 (*MsSPL10* and *14*), 9 (*MsSPL13*), 10 (*MsSPL6*, *7* and *16*), and 11 (*MsSPL2*) exons (Supplementary Figure 3). Moreover, we detected three conserved motifs among 22 MsSPL proteins. Motif I corresponded to the conserved SBP domain, which was commonly existed in all MsSPL proteins. In particular, MsSPL13 contained two independent SBP domains (Motif I). Motif II, a type of transmembrane region, was detected in MsSPL2, 6, and 16. Motif III was the conserved ANK domain, which presented in MsSPL2 and MsSPL16 (Supplementary Figure 4).

### Phylogenetic Analyses of the *SQUAMOSA Promoter-Binding Protein-Like* Gene Family

**FIGURE 2:**
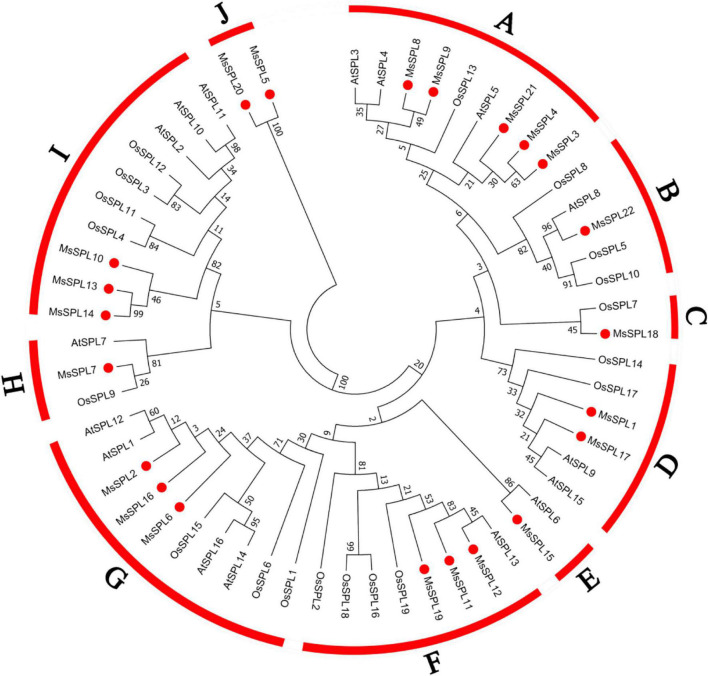
Phylogenetic relationships among *SPL* genes in alfalfa, *Arabidposis*, and rice. The phylogenetic tree was constructed using MEGA (Molecular Evolutionary Genetic Analysis) 7 based on ML (maximum likelihood) method; bootstrap was 1,000 replicates.

To evaluate the evolutionary relationships of plant *SPL* genes, we constructed the phylogenetic tree based on the alignment of amino acid sequences of 57 SPL proteins. The 57 SPL proteins included 22 MsSPL proteins as well as SPL proteins from the representative dicot *Arabidopsis* (16) and monocot rice (19). In the phylogenetic tree, the 57 SPL proteins were divided into ten clades: A (9 members), B (5), C (2), D (6), E (2), F (8), G (10), H (3), I (10), and J (2) (Figure 2). SPL proteins from dicotyledonous and monocotyledonous plants were found in all groups except for clade J, indicating that *SPL* genes existed before the divergence of dicots from monocots and then evolved independently. Moreover, clade J was consisted solely of two SPL proteins from alfalfa suggesting that *MsSPL5* and *MsSPL20* were alfalfa-specific *SPL* genes. These results demonstrate that *SPL* genes in alfalfa might have experienced gene duplication and functional differentiation within the genome (Figure 2).

Furthermore, we constructed another phylogenetic tree of *MsSPL* genes based on the alignment of the CDSs of 22 *MsSPL* genes followed by multiple sequence alignment via the neighbor-joining method in MEGA 7.0. The inferred evolutionary relationships of the 22 *MsSPL* genes were consistent with the results of the phylogenetic analysis of the 57 SPL proteins (Supplementary Figure 3). Taking the results of the gene structural analysis into additional consideration, and assuming that *SPL* genes with similar gene structures and conserved motifs have similar functions, we hypothesize that the specific motifs harbored by *SPL* genes are likely the main reason for the variations in functions observed among members of this gene family.

### Prediction and Validation of miRNA Cleavage Sites in *MsSPL* Genes

Since small RNAs and their targets are evolutionarily conserved among plant species, we predicted potential MREs in *MsSPL* genes using psRNATarget.^8^ As a result, 13 *MsSPL* genes were predicted to be degraded by *miR156*. Among these predicted targets, *MsSPL14* and *MsSPL18* showed one and two mismatches with the middle portion of *miR156a* sequence, respectively. Ten *MsSPL* genes (*MsSPL1*, *3*, *5*, *10–13*, *15*, *17*, and *20*) had sequences consistent with that of *miR156a*; and the remaining *MsSPL19* was predicted to be degraded by *miR156e* (Supplementary Table 3).

**FIGURE 3:**
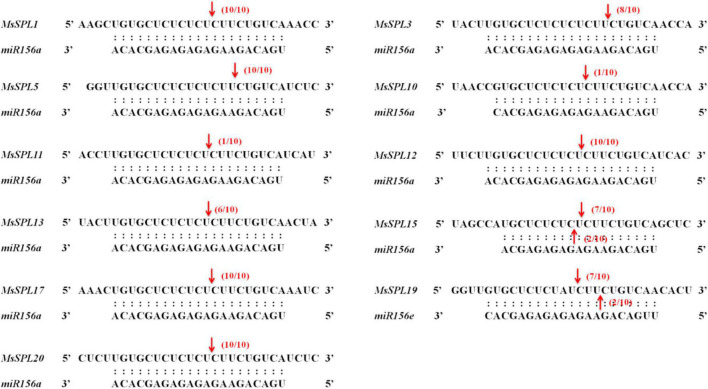
miR156 targets a group of *MsSPL* genes. The red arrows indicate the cleavage sites, and numbers below the arrows show the frequency of clones with matching 5′RACE product from this site out of total clones confirmed by sequencing.

To verify *in vitro* that *miR156* mediates the cleavage of its target *MsSPL* genes, we performed the RLM-5′-RACE assay modified for use in alfalfa. Total RNA was extracted from a mixture of alfalfa tissues, and gene-specific outer and inner primers were designed for 13 putative *miR156*-targeted *MsSPL* genes (Supplementary Table 3). After sequencing 10 positive clones obtained by nested PCR amplification, we determined that the 11 *MsSPL* genes with perfect matches to *miR156* could be degraded by *miR156* and that the cleavage sites were gene specific (Figure 3). In contrast, we were unable to detect the predicted *miR156* target cleavage fragments in *MsSPL14* and *MsSPL18*, which had one and two mismatches to *miR156*, respectively, suggesting that these two *SPL* genes are not degraded by *miR156*.

### Temporal and Spatial Expression Patterns of *MsSPL* Genes in Alfalfa

**FIGURE 4:**
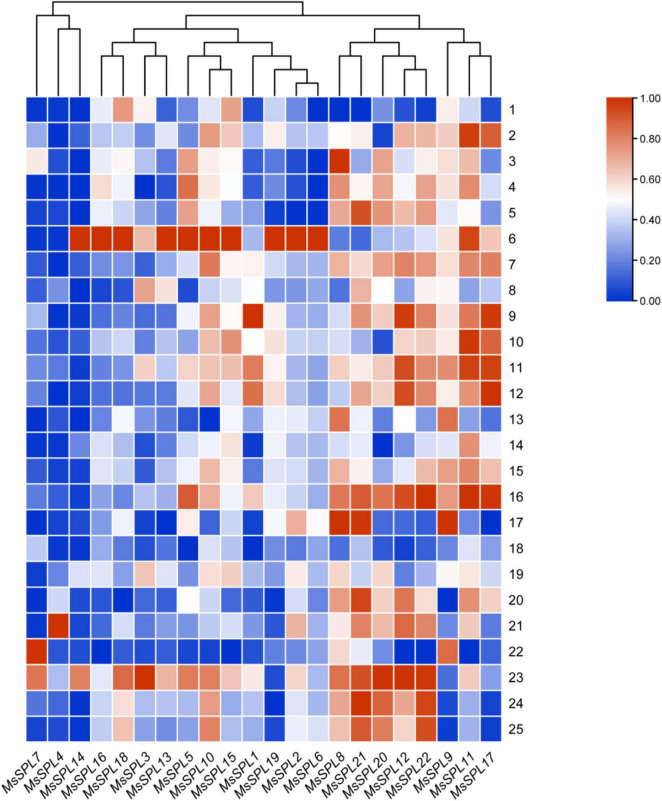
The spatiotemporal expression patterns of *MsSPL* genes. 1–25, respectively, represent various tissues of alfalfa, namely root, stem, neck, leaf, and head at seedling stage; root, stem, leaf, and head at re-greening stage; stem, stem node, head, and leaf at branching stage; stem, stem node, head, and leaf at squaring stage; stem, stem node, head, inflorescence, and leaf at flowering stage; seedpods at 0, 3, and 5 days after fertilization. The heatmap was constructed by relative expression data measured by qRT-PCR.

To clarify the roles of *MsSPL* genes in alfalfa growth and development, we used qRT-PCR to investigate the expression profiles of these genes in 25 different alfalfa tissues: stems, heads, and leaves at different developmental stages (seedling, re-greening, branching, squaring, and flowering stages); roots at seedling and re-greening stages; neck at the seedling stage; stem nodes at the branching, squaring and flowering stages; inflorescences at flowering stages; and seedpods at 0, 3 and 5 d after fertilization (Supplementary Table 4). According to the qRT-PCR analysis, all *MsSPL* genes were constitutively expressed in all 25 tissues with distinct expression patterns (Figure 4).

The expression patterns of these 22 *MsSPL* genes could be clustered into three types. In detail, three genes (*MsSPL4*, *7*, and *14*) were particularly highly expressed in one or two tissues, suggesting their specific roles in these tissues (Figure 4). Eight genes (*MsSPL8*, *9*, *11*, *12*, *17*, *20*, *21*, and *21*) were highly expressed in most tissues, and several of them showed consistently high expression during specific developmental stages. For instance, the transcript abundances of *MsSPL9*, *11*, and *17* exhibited high levels at seedling, branching, and squaring stages; while *MsSPL8*, *12*, *20*, *21*, and *22* exhibited significantly higher transcript abundances in tissues experiencing active cell proliferation, such as apical meristem tissue at the branching, squaring, flowering stages and seedpods at 0, 3, and 5 d after fertilization (Figure 4). The remaining 11 genes (*MsSPL1*, *2*, *3*, *5*, *6*, *10*, *13*, *15*, *16*, *18*, and *19*) showed another expression pattern, which was highly expressed in several tissues, but lowly expressed in most tissues. Among these genes, *MsSPL18* was predominantly expressed in leaves at different stages; interestingly, all of the genes had their highest transcription levels in roots at the re-greening stage (Figure 4). The various expression patterns of the 22 *MsSPL* genes suggest that they harbor multiple functions during alfalfa growth and development. In particular, the expression patterns of the two alfalfa-specific *SPL* genes were distinct. Among them, *MsSPL5* was highly expressed in heads at various stages, while *MsSPL20* was predominantly expressed in several tissues with active cell proliferation, suggesting the extensive function in alfalfa of the latter.

### Expression Profiles of *MsSPL* Genes in Response to Abiotic Stress

**FIGURE 5:**
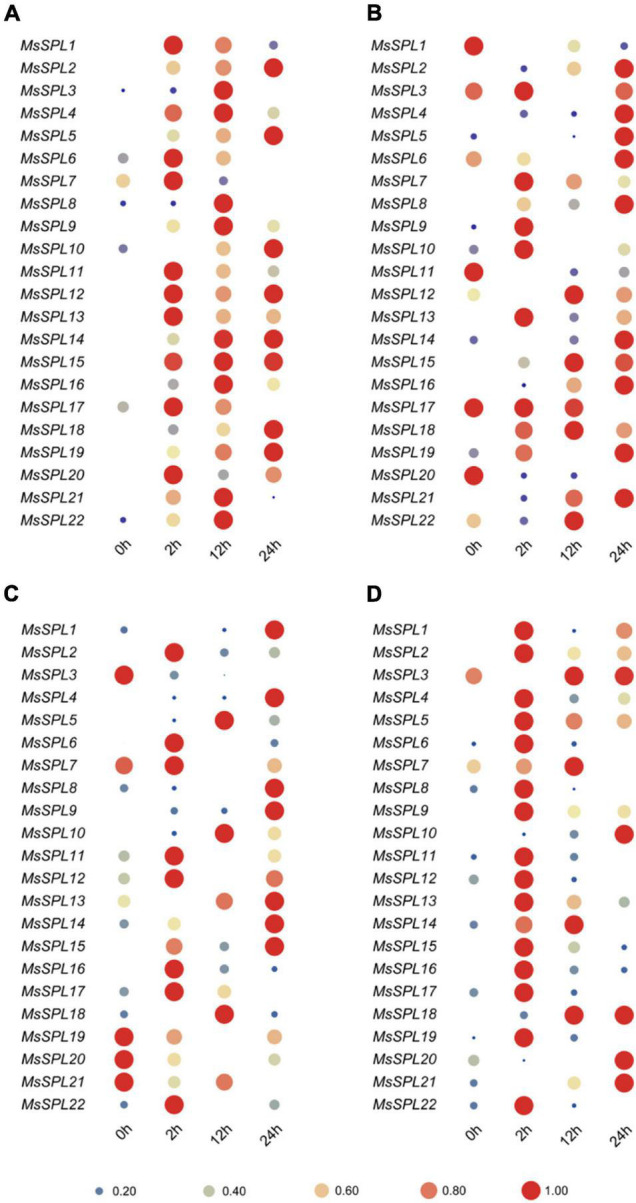
Expression of *MsSPL* genes in response to abiotic stress. Plants were treated with 0.3M NaCl **(A)**, 4**°**C **(B)**, 17% PEG **(C)**, and ABA **(D)**.

Abiotic stresses always induce gene expression to protect plant cells from abiotic injury. To decipher the roles of *MsSPL* genes in response to abiotic stresses, we analyzed the expression profiles of 22 *MsSPL* genes upon NaCl, 4°C and PEG treatment to simulate saline, cold, and drought conditions, respectively, as well as ABA treatment (Supplementary Table 4). All *SPL* genes except for *MsSPL5–7*, *10*, *17*, *19*, and *22* were dramatically up-regulated (more than three times) under NaCl treatment. Most of them (except for *MsSPL2*, *14*, and *18*) reached their peak transcript levels at 12 h. Moreover, the transcript abundances of *MsSPL1*, *13*, *14*, *20*, and *21* under salinity stress were more than 18 times higher than those in untreated alfalfa (Figure 5A). In regard to cold stress, 11 genes (*MsSPL1*, *2*, *4*, *5*, *6*, *9*, *10*, *13*, *14*, *15*, and *22*) were dramatically up-regulated (more than two times), while four genes (*MsSPL7*, *19*, *20*, and *21*) were down-regulated. Three genes (*MsSPL2*, *6*, and *22*) reached their peak transcript levels at 2 h, and six genes (*MsSPL1*, *4*, *9*, *13*, *14*, and *15*) had their maximum expression at 24 h after cold stress treatment (Figure 5B). Under PEG treatment, *MsSPL2*, *5*, *8*, *14*, and *21* were significantly sequentially up-regulated in 24 h, while *MsSPL1*, *3*, *20*, and *22* were significantly down-regulated. Additionally, *MsSPL7*, *9*, *10*, and *13* showed their peak transcript levels at 2 h, after which they decreased sharply; In particular, the expression level of *MsSPL14* was decreased at 2 h and dramatically increased at 24 h (Figure 5C). Under ABA treatment conditions, most *SPL* genes (*MsSPL1*, *2*, *4–6*, *8*, *9*, *11–13*, *15–17*, *19*, and *22*) reached their maximum transcript levels at 2 h and then decreased. In contrast, two genes (*MsSPL10* and *18*) showed steadily increased expression for 12 h after treatment, while two genes (*MsSPL20* and *21*) were significantly decreased at 2 h and then increased sharply or slightly (Figure 5D).

In addition, we compared the significant changes of *SPL* genes induced by NaCl, PEG, and ABA treatment. We found that several critical *SPL* genes (*MsSPL2*, *8*, *13*, and *15*) were simultaneously induced by salinity, drought, and ABA. Five (*MsSPL2*, *8*, *13*, *15*, and *21*), four (*MsSPL2*, *8*, *13*, and *15*), and twelve (*MsSPL1*, *2*, *4*, *8*, *9*, *11*, *12*, *13*, *14*, *15*, *16*, and *22*) genes were induced by various combinations of two of these stresses (Supplementary Figure 5). Moreover, different stresses induced unique *SPL* genes. For instance, *MsSPL3*, *18*, and *20* were only induced by salinity; *MsSPL7* was only induced by drought; and *MsSPL5*, *6*, *17*, and *19* were only induced by ABA treatment (Supplementary Figure 5). The significantly different transcript abundances of *MsSPL* genes in response to abiotic stress suggest their vital regulatory roles in the prevention of abiotic injury in alfalfa.

### Response Patterns of *MsSPL* Genes to Different Plant Hormones

**FIGURE 6:**
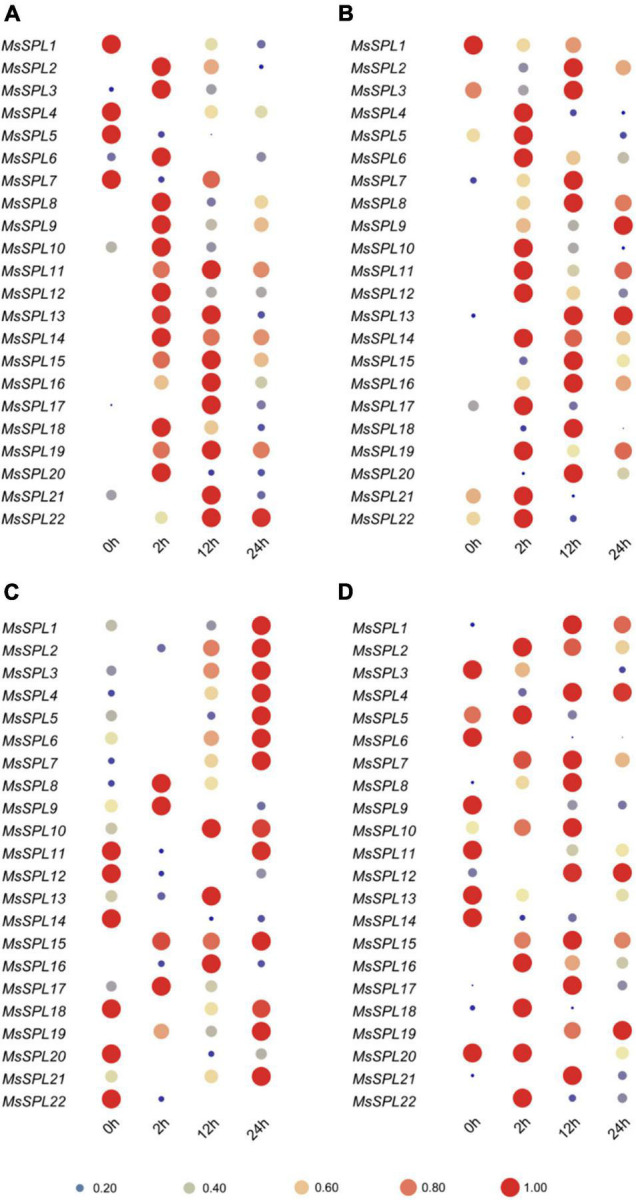
Expression of *MsSPL* genes in response to hormones. Plants were watered with 0.3M GA **(A)**, GR24 **(B)**, IAA **(C)**, and MeJA **(D)**.

Plant hormones extensively participate in a variety of plant growth and developmental processes. Studying the expression patterns of *MsSPL* genes under hormone treatments should thus help elucidate the functions of *MsSPL* genes. We investigated the transcript abundances of *MsSPL* genes in response to GA3, GR24, IAA, and MeJA treatment (Supplementary Table 4). We found that 19 (*MsSPL1–3*, *6–9*, *11–21*, and *22*) and 2 (*MsSPL4* and *5*) *SPL* genes were significantly up- and down-regulated, respectively, in 12 h after treatment with GA3. Specifically, the transcript abundances of five genes (*MsSPL8, 9, 12, 14*, and *20*) at 2 h were more than 7 times higher than those in untreated alfalfa (Figure 6A). As for the GR24 treatment, the transcript levels of 16 genes (*MsSPL2*, *4*, *6–8*, *10–19*, and *20*) reached their peak in 12 h; and the expression of three genes (*MsSPL4*, *9*, and *10*) were significantly increased (more than 9 times) after GR24 treatment. Additionally, four genes (*MsSPL1*, *5*, *21*, and *22*) were significantly down-regulated at 24 h (Figure 6B). In IAA-treated alfalfa, transcript abundances of nine genes (*MsSPL1*, *3–7*, *10*, *13*, and *21*) were decreased at 2 h and then steadily increased to their maximum levels at 24 h; while four genes (*MsSPL8*, *9*, *15*, and *19*) reached their peak expression at 2 h after treatment. In addition, the expression levels of *MsSPL14*, *18*, *20*, and *22* were significantly decreased after IAA treatment (Figure 6C). Under MeJA treatment condition, ten genes (*MsSPL1*, *2*, *4*, *7*, *8*, *15*–*17*, *19*, and *21*) and six genes (*MsSPL5, 6, 9, 13, 14*, and *20*) were dramatically up- and down-regulated (more than two times). In particular, five genes (*MsSPL4*, *7*, *8*, *15*, and *21*) had transcript levels four times higher than control, and the transcript levels of others were slightly changed (Figure 6D). These results demonstrate that *MsSPL* genes are involved in the plant hormone regulatory network that controls alfalfa growth and development.

### Overexpression of *MsSPL20* Delayed Flowering Time in Alfalfa

According to the above results from phylogenetic analysis and expression profiles in various tissues, we hypothesized that *MsSPL20* might be involved in an alfalfa-specific regulation pathway. Thus *MsSPL20* was selected as the target for further gene functional research. We obtained six clonally transgenic alfalfa plants over-expressing *MsSPL20* via *Agrobacterium*-mediated transformation. The qRT-PCR analysis showed that the transcript levels of *MsSPL20* in six transgenic alfalfa plants were 90 (OE1), 47 (OE2), 38 (OE3), 18 (OE4), 15 (OE8), and 58 (OE9) times greater than that in control. Among them, OE1 and OE9 consistently showed 5–10 days delayed in flowering time compared with control plants. Furthermore, we measured the biomass of OE1 and OE9 by clipping them when they were at the early bloom stage. The results showed that the transgenic plants exhibited significantly 17–21% increased biomass compared with control plants. In addition, the forage quality was also determined and both plants showed no significant differences (Supplementary Figure 6).

**FIGURE 7:**
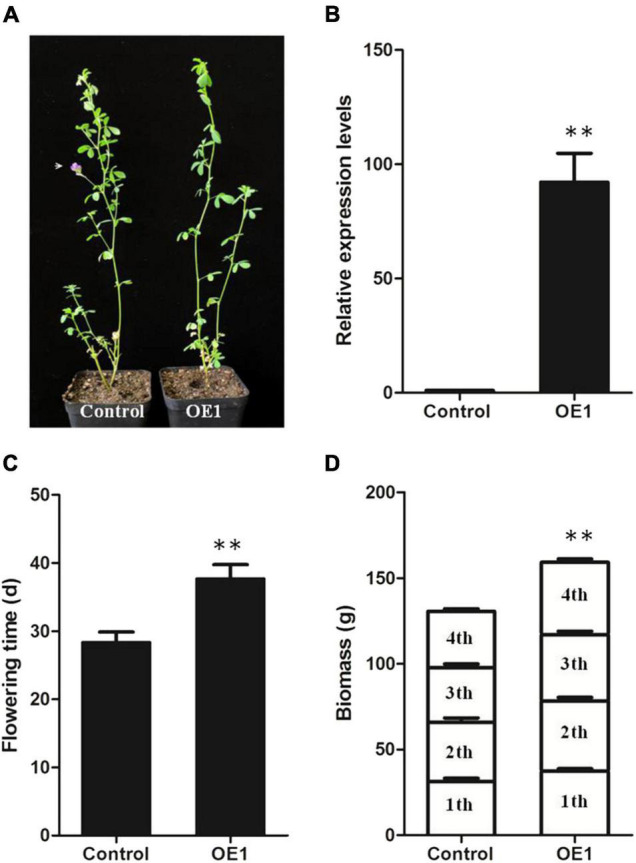
Transgenic alfalfa showing delaying flowering time and increased biomass. **(A)** The phenotype of control and OE1. **(B)** *MsSPL20* relative expression levels. **(C)** Flowering time of control and OE1. **(D)** Biomass of control and OE1. ** indicates significant difference at *P* < 0.01.

To perform further characterization, OE1, which showed a more than 90-fold increase in *MsSPL20* transcript levels was propagated using shoot cuttings. These propagated transgenic plants exhibited significant delays in the flowering time of 7–10 days (Figures 7A–C). Moreover, we calculated the biomass of transgenic and control plants by clipping them four times once they were flowering to simulate the biomass in a harvest season in a year. The results indicated that the biomass of the transgenic plants was significantly higher than that of the control plants by approximately 20%, as was their dry biomass (Figure 7D and Supplementary Table 4). The biomass data collection time for control plant was 125 days, which was much shorter than that for the transgenic lines (157 days). Moreover, we compared the biomass from each clipping and found that the individual clipping contributed to 21.3, 22.6, 22.8, and 33.3% increased biomass, respectively. These results suggest that the increased biomass mostly results from the longer vegetative growth period. Unsurprisingly, the forage quality was not significantly different between control plants and transgenic plants (Supplementary Table 5). These results strongly demonstrate that *MsSPL20* regulates flowering time and forage biomass yield without affecting forage quality in alfalfa.

### *MsSPL20* Regulates Flowering Time by Delaying Floret Development

**FIGURE 8:**
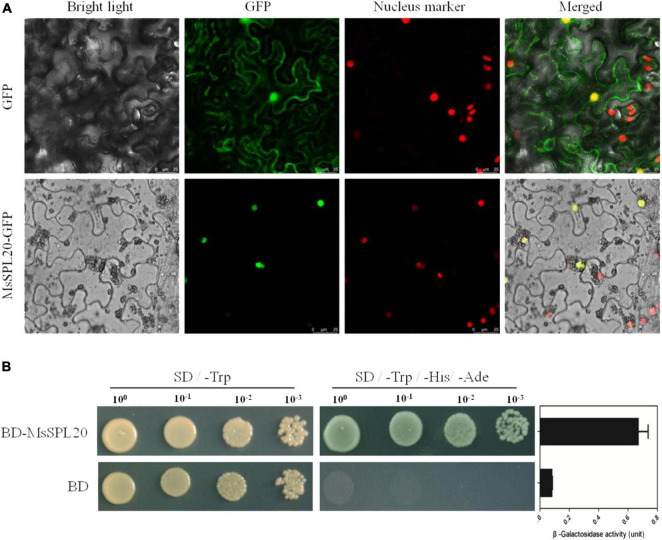
Molecular characterization of *MsSPL20*. **(A)** Sub-cellular localization in tobacco epidermal cells. Scale bars, 25 μm. **(B)** *Trans*-activation activity assay in yeast. BD, GAL4 DNA binding domain.

As the typical transcriptional factor, SPL proteins located in nucleus and bind to *cis-*elements (GTAC box) in the promoters of downstream genes to regulate their transcription (Birkenbihl et al., 2005). The sub-cellular localization analysis in tobacco epidermal cells showed that MsSPL20-GFP accumulated only in nucleus, whereas the GFP alone was present throughout the cell, indicating that *MsSPL20* functions in nucleus (Figure 8A). The *trans*-activation activity assays showed that MsSPL20 possessed strong transcriptional activation activity in yeast, suggesting its potential roles in downstream gene regulation (Figure 8B). To further elucidate the molecular basis of *MsSPL20* in alfalfa flowering time regulation, we conducted RNA-seq and deeply studied the differentially expressed genes (DEGs) between control and transgenic plants. OE1, which showed 90 times higher *MsSPL20* transcript levels than control plants, was used for RNA-seq. The comparative transcriptome analysis revealed that 129 and 342 genes were respectively, up- and down-regulated in transgenic plants relative to control plants. Moreover, the change degree of down-regulation genes was much higher than that of up-regulation genes (Supplementary Figure 7). GO analysis demonstrated that DEGs were enriched in terms including developmental process, cellular component assembly, pollen development, gametophyte development, etc., implying that *MsSPL20* was involved in floral organ development (Supplementary Figure 8).

If these DEGs are indeed the direct targets of *MsSPL20*, their promoters should contain the conserved GTAC box. In support of our hypothesis, we scanned the promoters of DEGs and found that the conserved *cis-*elements (GTAC box) were existed in the promoters of almost all of the DEGs (471). Among these DEGs, 38 were predicted to be involved in floret development (Supplementary Table 6). Twelve DEGs were selected for further qRT-PCR analysis, and the results showed that the transcript levels of the 12 genes were consistent with the RNA-seq results, indicating the reliability of transcriptome profiles (Supplementary Figure 9 and Supplementary Table 7). Two florigen related genes, *HD3A* (*Heading date 3A*, *Ms.gene51913*, the homolog of *FT*) and *FTIP1* (*FT-INTERACTION PROTEIN 1*, *Ms.gene017959*, the essential regulator for florigen transport), were down-regulated in transgenic plants, suggesting the delaying formation and transportation of florigen, which is the most important signal of floret development in plants (Komiya et al., 2008; Liu et al., 2012). In addition, several flowering delay factors were up-regulated in transgenic plants. For instance, *Ms.gene005871* (*TEMPRANILLO 1*, *TEM1*) was reported to repress the production of FT and gibberellins, leading to delaying in flowering time (Hu et al., 2021); *Ms.gene055550* (*HASTY-like Protein 1*, *HST1*) extends the vegetative phase by repressing the FPIs, also resulting to delaying flowering (Matsoukas et al., 2013). Overall, these results suggested that *MsSPL20* might regulate alfalfa flowering time by regulating gene involved in floret development regulation through directly binding to the promoters of floret development-related factors.

## Discussion

### Functional Diversity of *SQUAMOSA Promoter-Binding Protein-Like* Family Genes in Plants

As an extensively elucidated gene family, *SPL* genes are widespread in all green plants, including algae, mosses, gymnosperms, and angiosperms (Preston and Hileman, 2013). More and more studies demonstrated that *SPL* genes participate in a broad range of plant biological processes (Chen et al., 2010; Wei et al., 2018). In Arabidopsis, there are *16 SPL* genes which groups into two subfamilies based on their size and sequence similarity (Guo et al., 2008). The large group consists of 5 *SPL* genes (*SPL1*, *SPL6*, *SPL12*, *SPL14*, and *SPL16*), whereas, the remaining 11 *SPL* are addressed as the small group (Xing et al., 2010). Except *SPL8*, *SPL* genes in small group could be targeted by *miR156*; and showed multiply functions in plants, such as developmental phase transition, shooting branching, anthocyanin biosynthesis, abiotic stress tolerance, etc. (Gou et al., 2011; Cui et al., 2015; Zhang et al., 2015). *SPL* genes in large group are proved to participate in plant thermotolerance, innate immunity, architecture regulation, etc. (Padmanabhan et al., 2013; Chao et al., 2017).

Evidence is increasing that *SPL* genes are multifunctional in plant growth and development and showed potential application for crop genetic modification (Wang and Wang, 2015). In rice, *SPL* genes associated with tiller/branching number (*OsSPL7* and *OsSPL14*), plant height (*OsSPL7*), grain number (*OsSPL2* and *OsSPL17*), grain size (*OsSPL13* and *OsSPL16*), heading date, and grain quality (*OsSPL16*) have been identified; and their molecular mechanisms have been extensively illustrated (Jiao et al., 2010; Miura et al., 2010; Wang S. et al., 2012; Si et al., 2016; Yue et al., 2017; Dai et al., 2018). In maize, *SPL* genes are proved to regulate flowering time (*ZmSPL25*), plant/ear height and tiller (*UB2* and *UB3*), leaf angle (*LG1*), tassel and ear architecture (*UB3* and *LG1*), and grain size and shape (*TGA1*) (Chuck et al., 2014; Du et al., 2017; Wei et al., 2018). Additionally, *SPL* genes also play vital roles in the regulation of plant biotic and abiotic stress tolerance. The *Nicotiana SPL* gene *NbSPL6* is essential for N-mediated resistance to *tobacco mosaic virus*, and its *Arabidopsis* ortholog, *AtSPL6*, is required for TIR-NB-LRR-mediated resistance against *Pseudomonas syringae* carrying the *avrRps4* effector (Padmanabhan et al., 2013).

In our study, we identified 22 *MsSPL* genes in alfalfa genome and conducted phylogenetic analysis of SPL protein to investigate the evolutionary relationships of *SPL* genes (Figures 1, 2). Interestingly, we found that each *AtSPL* gene had one or three orthologous genes in alfalfa, as well as two alfalfa-specific *SPL* genes (*MsSPL5* and *MsSPL20*), indicating the gene duplication in this species. Combined with the results from RLM-5′-RACE, we noticed possible gene functional differentiation between orthologous genes. Evidence came from the different post-transcriptional regulation between *SPL3/SPL4* and *MsSPL8/9*. *AtSPL3*, and *AtSPL4* are targeted by *miR156*, whereas their orthologous genes in alfalfa (*MsSPL8* and *MsSPL9*) cannot be targeted by miR156 (Figure 3). Previous researches indicated that *SPL* genes in the same clade had similar functions. For instance, *AtSPL13*, *OsSPL16*, and *MsSPL12* in clade F were proved to participate in flowering time regulation in different plants (Martin et al., 2010; Wang S. et al., 2012; Gao et al., 2018). Nevertheless, we cannot always predict the function of an *SPL* gene based on its orthologous gene, as neo-functionalization is widespread during speciation. For example, *AtSPL3* in clade A primarily promotes floral induction and/or floral meristem identity; however, its ortholog in rice, *OsSPL13*, positively regulates grain size by influencing cell proliferation (Cardon et al., 1999; Si et al., 2016). Given the diverse functions of the *SPL* genes in plants, the biological function of *SPL* genes in alfalfa should thus be explored individually.

Furthermore, the alfalfa-specific *SPL* gene, *MsSPL20*, was deeply functional characterized. Transgenic alfalfa plants over-expressing *MsSPL20* showed delayed flowering time and increased biomass without affecting forage quality (Supplementary Figure 6 and Supplementary Table 5). Further researches showed that the majority effect on biomass was caused by the extended vegetative growth period (Figure 7). The subsequent transcriptome analysis illustrated that *MsSPL20* delayed flowering time by regulating the expression of genes involved in floret development, such as *HD3A*, *FTIP1*, *TEM1*, and *HST1*. Among these four candidate genes, the expression of *HD3A* and *FTIP1* were down-regulated; while, *TEM1* and *HST1* were up-regulated in transgenic plants (Supplementary Figure 9). Previous studies have shown that the first two genes positively regulate flowering time, and the regulations of the latter two are negative (Komiya et al., 2008; Liu et al., 2012; Matsoukas et al., 2013; Hu et al., 2021). Since several GTAC-box exists in the promoters of these four genes, we inferred that MsSPL20 could directly bind to these promoters to regulate flowering time in alfalfa, which should be validated in further study.

### Potential Application of *MsSPL* Genes in Alfalfa Molecular Breeding

Over the past two decades, candidate gene-related markers for molecular marker-assisted breeding have been rapidly developed in major crops, and the concept of breeding by design has gradually become reality (Peleman and van der Voort, 2003; Xu et al., 2021). The rice cultivar Zhongke 804, which possesses several favorable alleles (e.g., *IPA4*, Q*sw5*, *GS3*, *Ghd8*, *TAC1*, *SSII-1*, *DEP1*, and *SBE1*) promoting the ideal plant architecture, high yield, superior quality, and strong resistance to rice blast disease, has recently become popular in northern China (Zeng et al., 2017; Li et al., 2019). This successful application of molecular-designed breeding in rice also demonstrates that rational design is a powerful strategy for meeting the challenges of future crop breeding, particularly the pyramiding of multiple complex traits. Given that alfalfa is the most important and widely planted forage crop in the world, the exploration of candidate genes for molecular marker-assisted breeding is urgently needed to meet the increased demands associated with this crop.

During the past few years, *SPL* genes have been shown to influence forage biomass by controlling shoot branching, delaying flowering time, and increasing tolerance to abiotic stresses. The transgenic alfalfa over-expressing *miR156* exhibits elevated biomass and improved drought tolerance resulting from the down-regulation of three *SPL* genes, indicating that *miR156-SPL* module is a promising tool for alfalfa improvement (Aung et al., 2015). Transgenic alfalfa silencing *MsSPL13* displays more lateral branches and delayed flowering time (Gao et al., 2018). Additionally, Over-expression of *MsSPL8* inhibits branching by suppressing axillary bud formation, and down-regulation of *MsSPL8* has been found to enhance salt and drought tolerance in alfalfa (Gou et al., 2018). These studies have revealed the vital roles of known *SPL* genes in forage biomass development and stress tolerance.

In this study, the expression profiling of *MsSPL* genes, including their temporal and spatial expression patterns and response to abiotic stresses and various phytohormones, provided a referable basis for *MsSPL* gene functional research in alfalfa (Figures 4–6). For instance, the expression of *MsSPL12* (*SPL13* described by Gao et al., 2018) was significantly elevated after treatment with GR24, an important hormone in plant branching, and silencing of this gene resulted in an increased number of lateral branches. Moreover, the expression level of *MsSPL22* (*SPL8* by Gou et al., 2018) was significantly changed under abiotic stress. Functional research on this gene suggested that the down-regulation of *SPL8* expression improved abiotic stress tolerance in alfalfa. The transcript abundances of the previously reported genes in our study explained the observed phenotypes of transgenic plants to some extent; therefore, we can infer the functions of *MsSPL* genes in alfalfa from the expression profiling we recorded.

To verify the hypothesis, *MsSPL20* (an alfalfa-specific *SPL* gene), which is predominantly expressed predominantly in several tissues with active cell proliferation, was selected as a candidate gene for functional analysis. As expected, transgenic alfalfa over-expressing *MsSPL20* altered flowering time and biomass (Figure 7). Nevertheless, some *MsSPL* genes with markedly changed transcript abundances in our study remain to be investigated. For example, the expression levels of *MsSPL1, 6, 7, 11, 12*, *13*, *15*, and *17* were sharply increased under NaCl treatment, suggesting that these genes might be involved in alfalfa salinity tolerance regulation (Figure 5). Expression profiling of *MsSPL* genes in response to abiotic stresses is an effective tool for the application of these genes to alfalfa abiotic stresses tolerance breeding. In addition, the transcript abundances of *MsSPL6*, *10*, and *13*, which peaked at the branching stage, were significantly changed under GA3 and GR24 treatments could be investigated as the candidate genes of alfalfa branching regulation (Figures 4, 6). Overall, the expression profiling of *MsSPL* genes in alfalfa provided the basic perspective on the biological functions of *MsSPL* genes; and has supplied several candidate *SPL* genes for alfalfa high-yield and abiotic stresses tolerance breeding.

## Conclusion

In conclusion, we charactered 22 *MsSPL* genes in alfalfa genome and found that 11 *MsSPL* genes with perfect matches to miRNA response elements (MREs) could be degraded by *miR156*. Meanwhile, we investigated the temporal and spatial expression patterns of *MsSPL* genes and their expression profiling in response to multiple treatments. More importantly, a candidate gene, *MsSPL20*, was proved to delay flowering time and increase biomass by regulating genes involved in floret development. This study provides valuable information for future research of *SPL* genes in alfalfa and supplies candidate genes for alfalfa molecular-assisted breeding utilization.

## Data Availability Statement

The original contributions presented in this study are included in the article/Supplementary Material, further inquiries can be directed to the corresponding authors. The raw data obtained by RNA-seq were deposited in the short read archive (SRA) databank and are available under the accession number PRJNA773924 (https://www.ncbi.nlm.nih.gov/bioproject/PRJNA773924/).

## Author Contributions

LM, XW, and YP conceived and designed the experiments. LM, WL, and XL performed the experiments. HW and YZ performed the transformation assays. LM and XL processed the data analysis. LM drafted the manuscript. XW and YP reviewed the manuscript. All authors approved the final version of the study.

## Conflict of Interest

The authors declare that the research was conducted in the absence of any commercial or financial relationships that could be construed as a potential conflict of interest.

## Publisher’s Note

All claims expressed in this article are solely those of the authors and do not necessarily represent those of their affiliated organizations, or those of the publisher, the editors and the reviewers. Any product that may be evaluated in this article, or claim that may be made by its manufacturer, is not guaranteed or endorsed by the publisher.

## Footnotes

1
https://alfalfatoolbox.org/blast/

2
http://smart.embl-heidelberg.de/

3
http://web.expasy.org/compute_pi/

4
http://gsds.cbi.pku.edu.cn/

5
http://mg2c.iask.in/mg2c_v2.0/

6
https://megasoftware.net

7
http://meme-suite.org/tools/meme

8
http://plantgrn.noble.org/psRNATarget/
