# Supplementary figures and images for: Characterization of Squamosa-Promoter Binding Protein-Box Family Genes Reveals the Critical Role of MsSPL20 in Alfalfa Flowering Time Regulation
Source: Front Plant Sci. 2022 Jan 5;12:775690. doi: 10.3389/fpls.2021.775690 (PMC8766856; doi:10.3389/fpls.2021.775690)

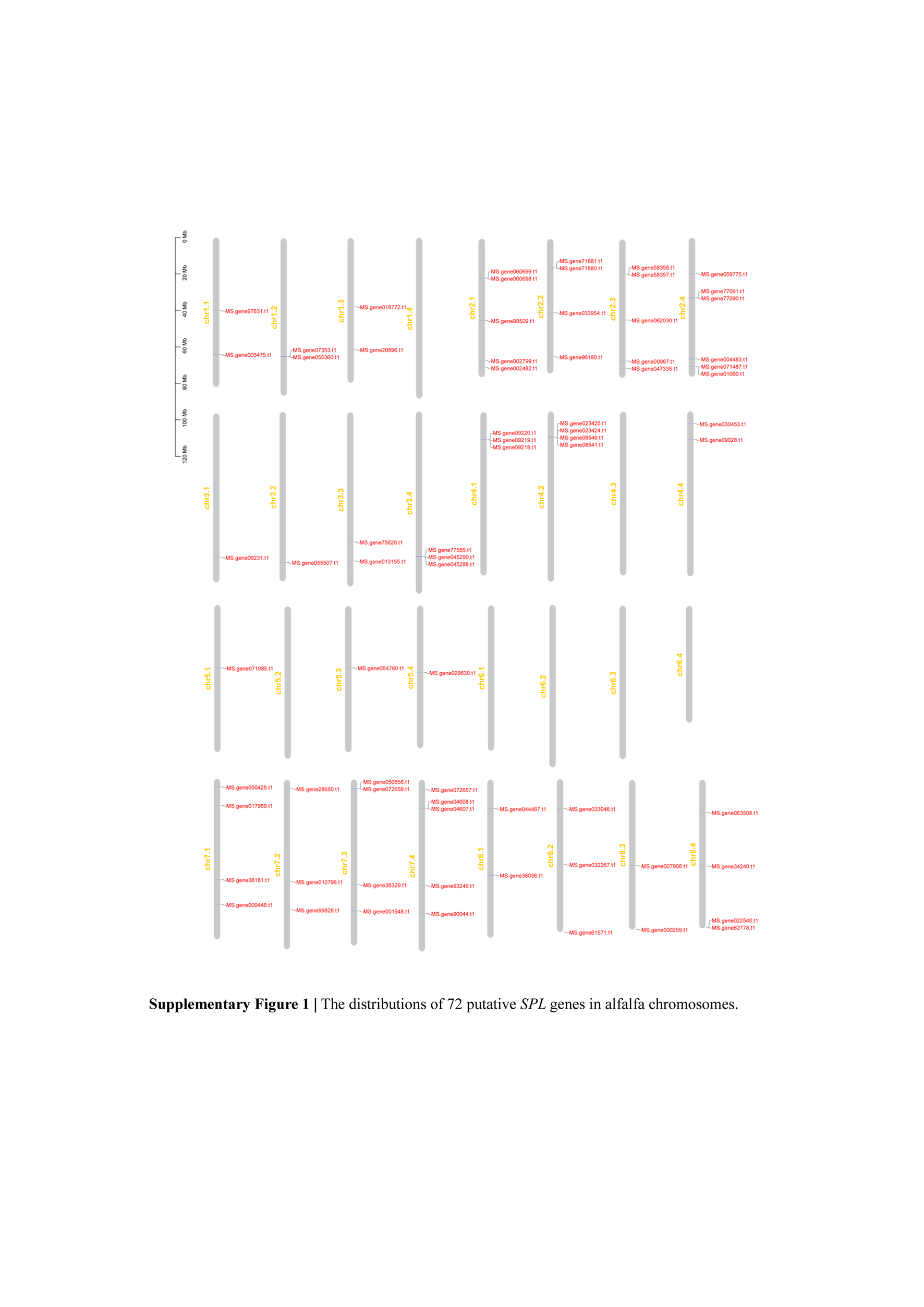

Supplement: Supplementary file 1 [file Image_1.TIF]

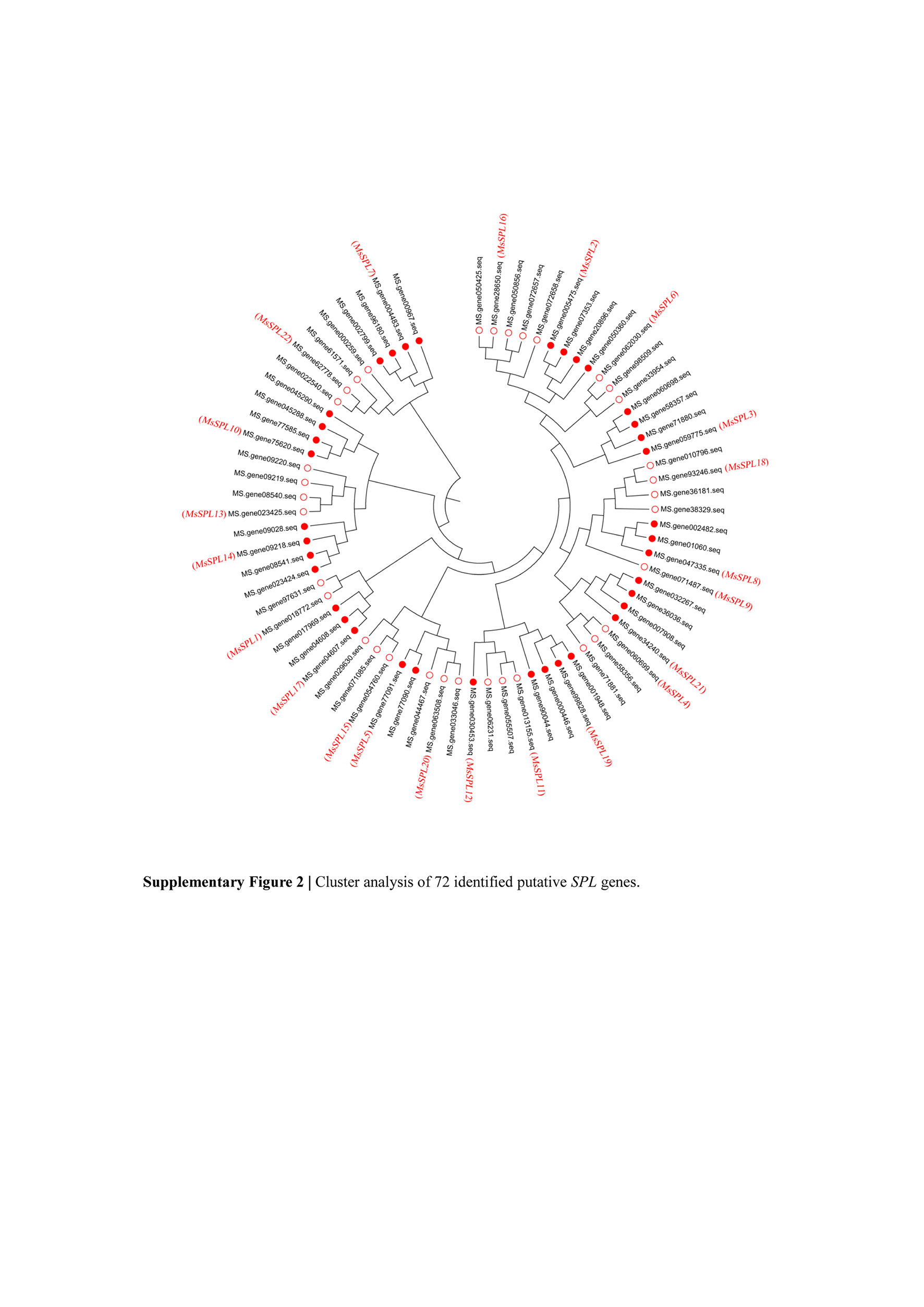

Supplement: Supplementary file 2 [file Image_2.TIF]

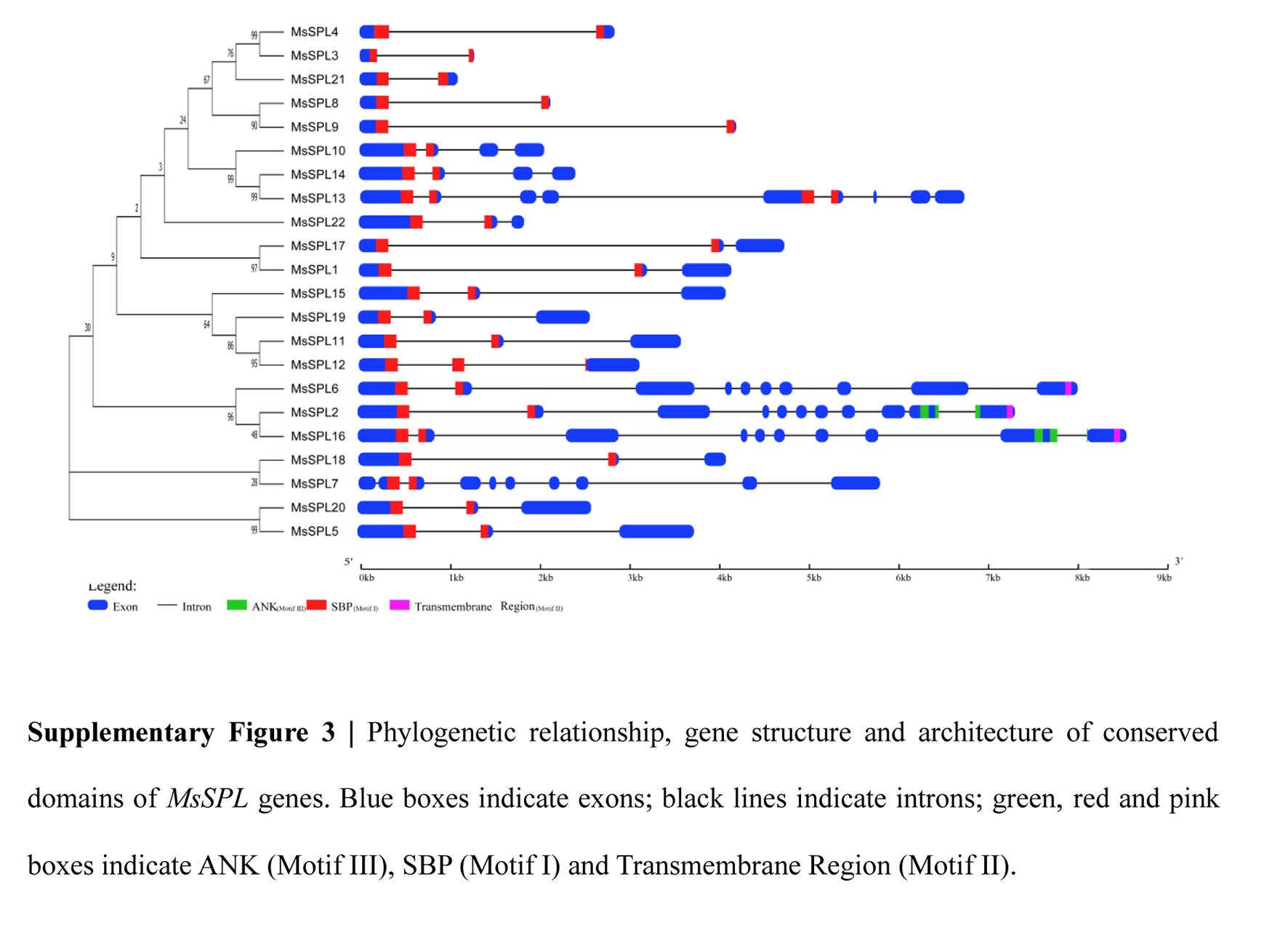

Supplement: Supplementary file 3 [file Image_3.TIF]

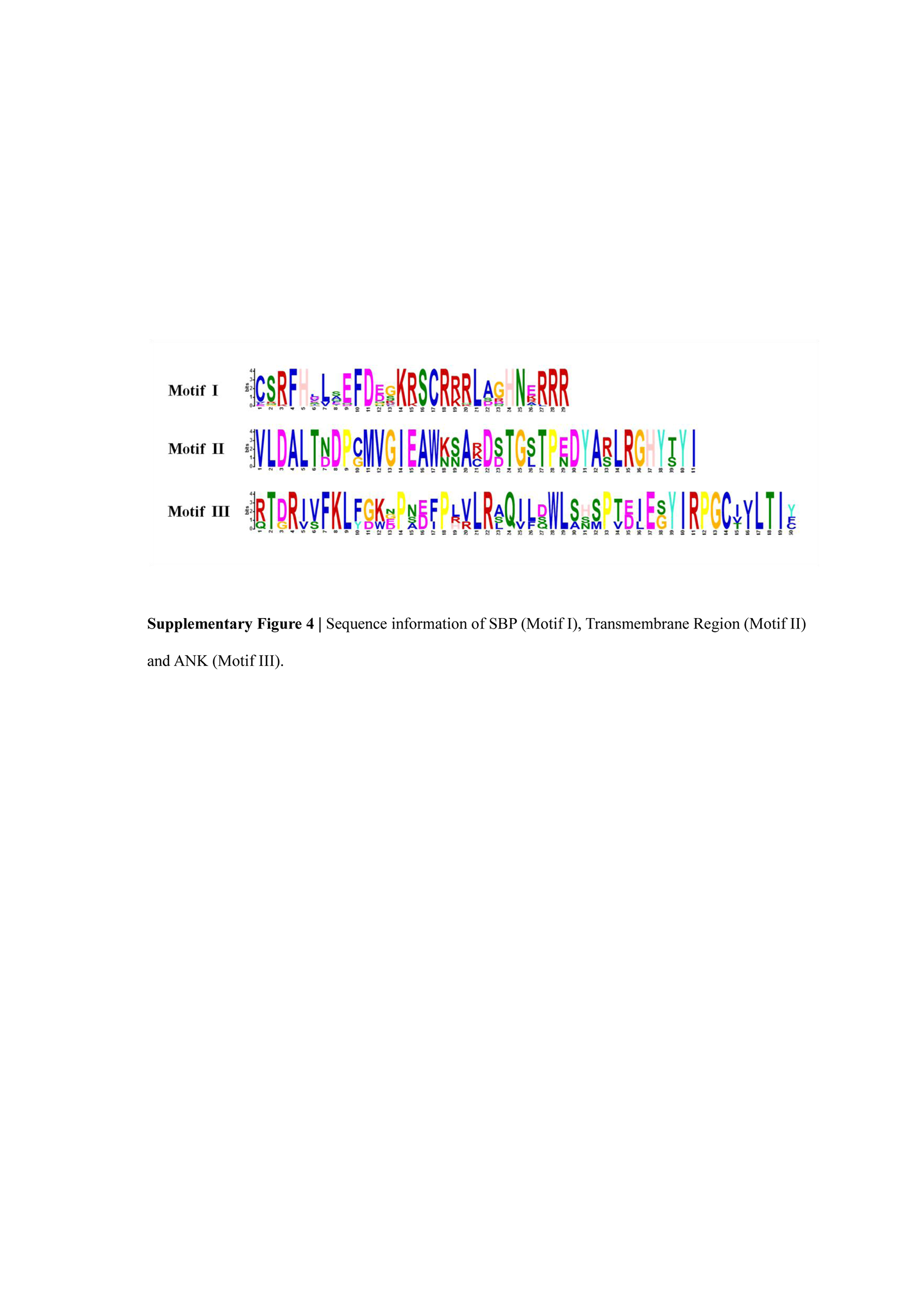

Supplement: Supplementary file 4 [file Image_4.TIF]

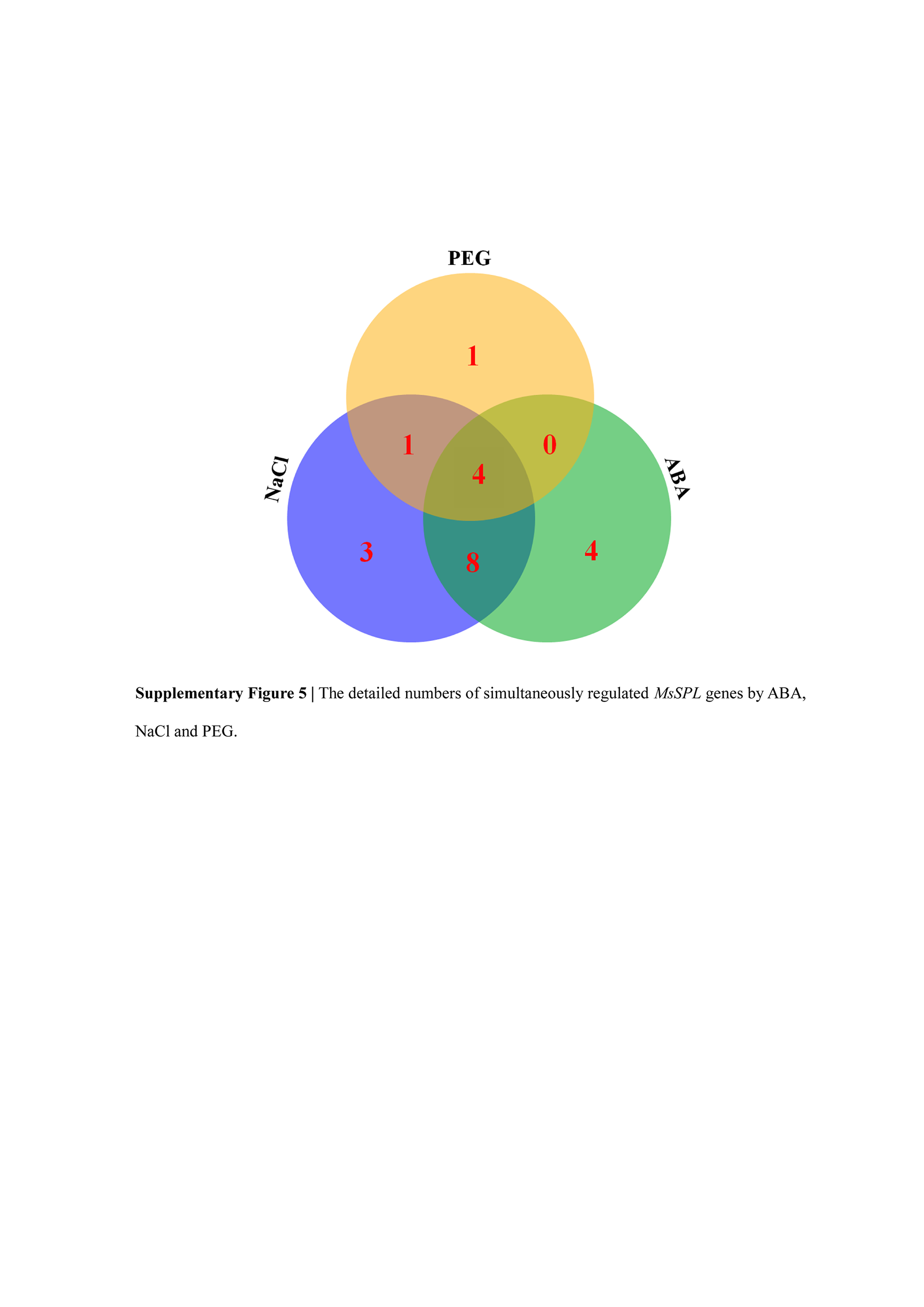

Supplement: Supplementary file 5 [file Image_5.TIF]

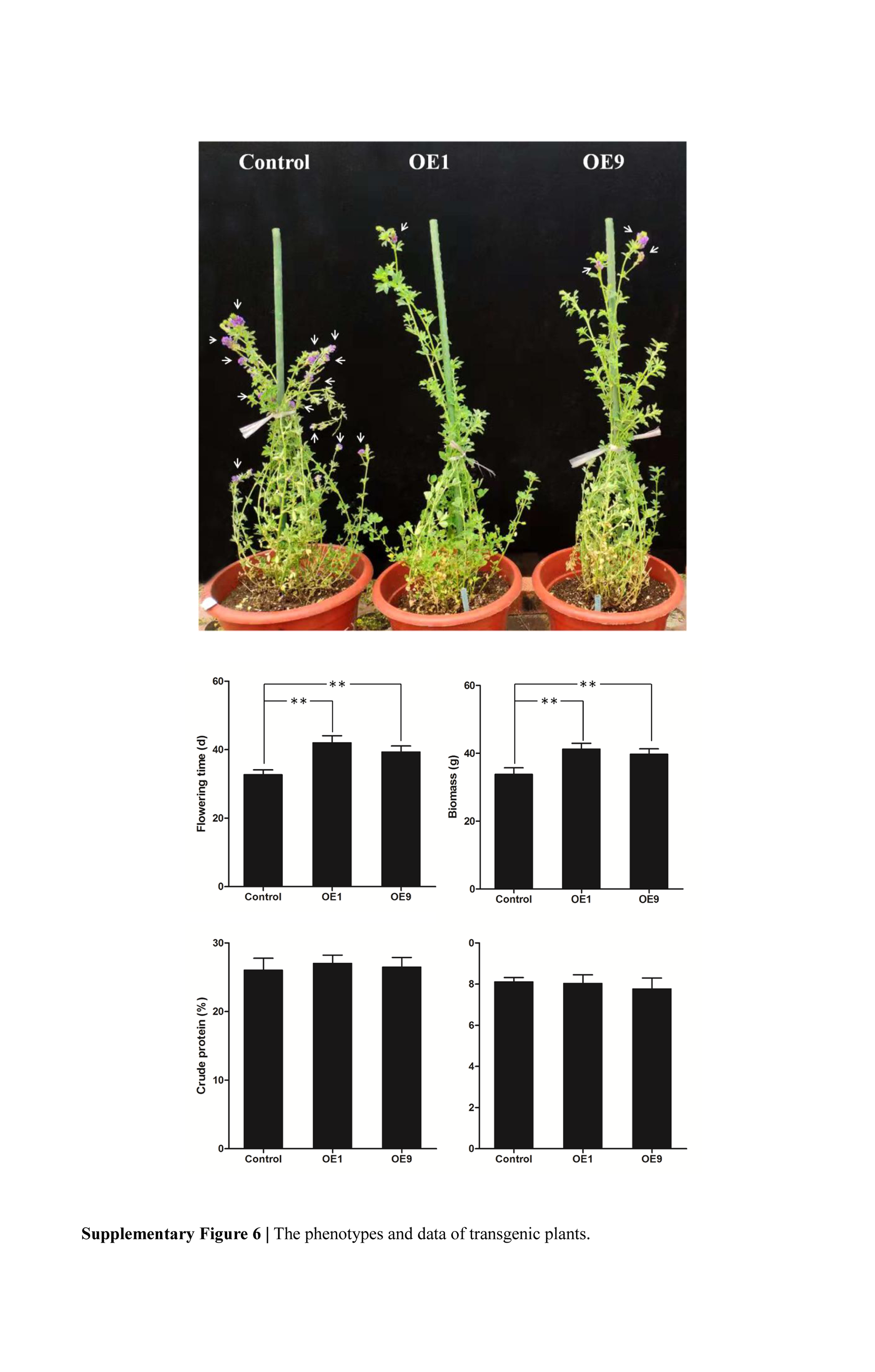

Supplement: Supplementary file 6 [file Image_6.TIF]

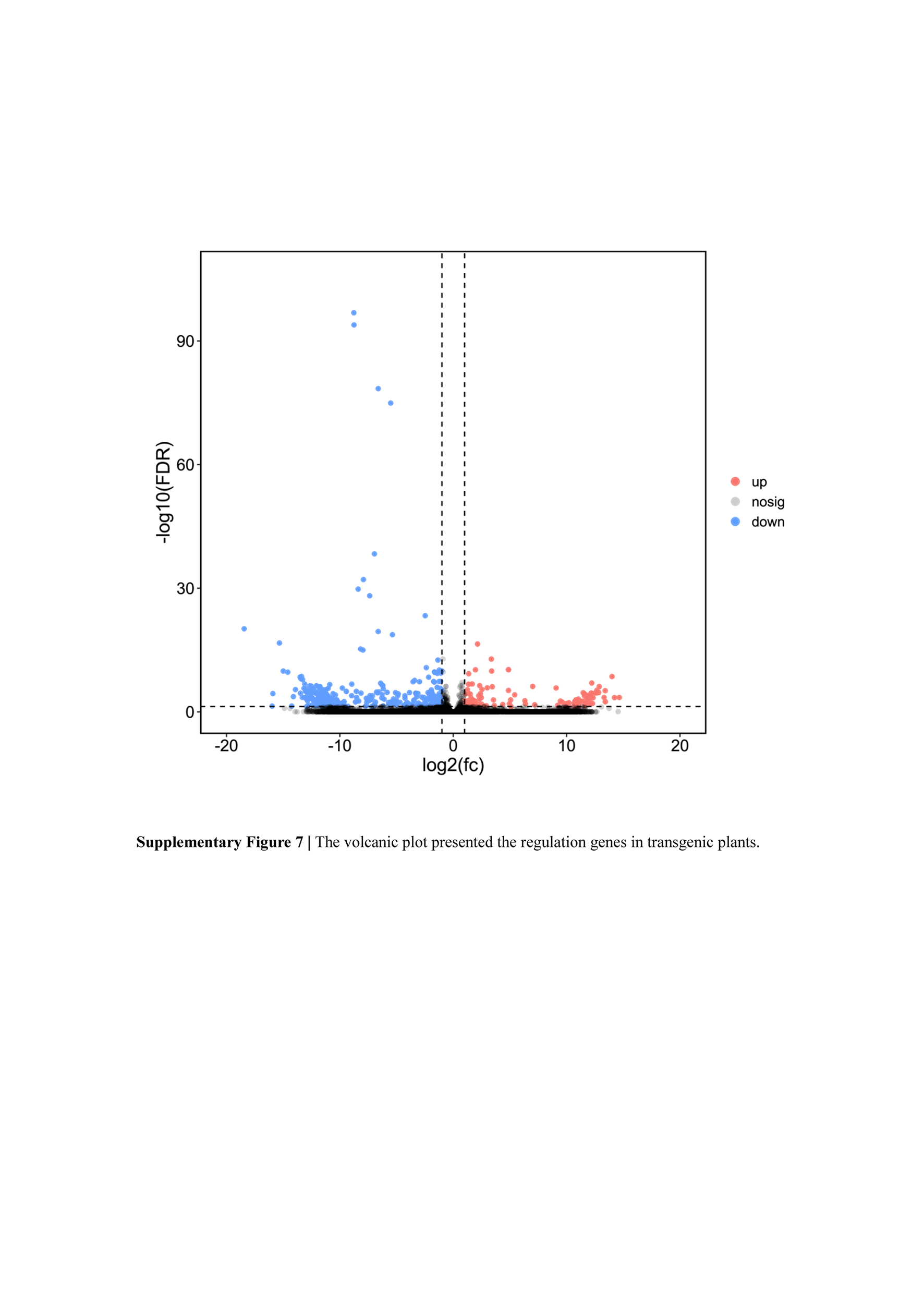

Supplement: Supplementary file 7 [file Image_7.TIF]

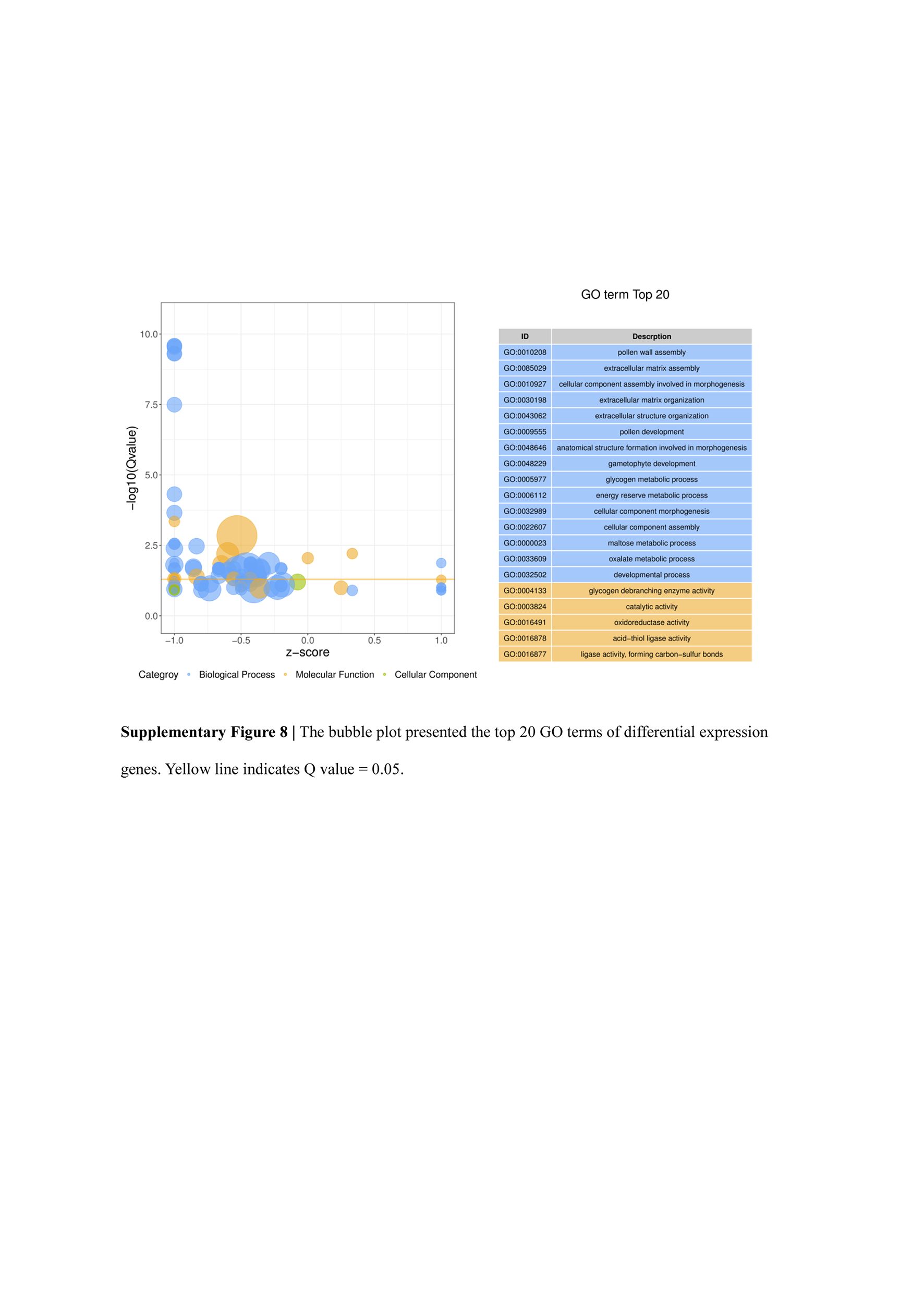

Supplement: Supplementary file 8 [file Image_8.TIF]

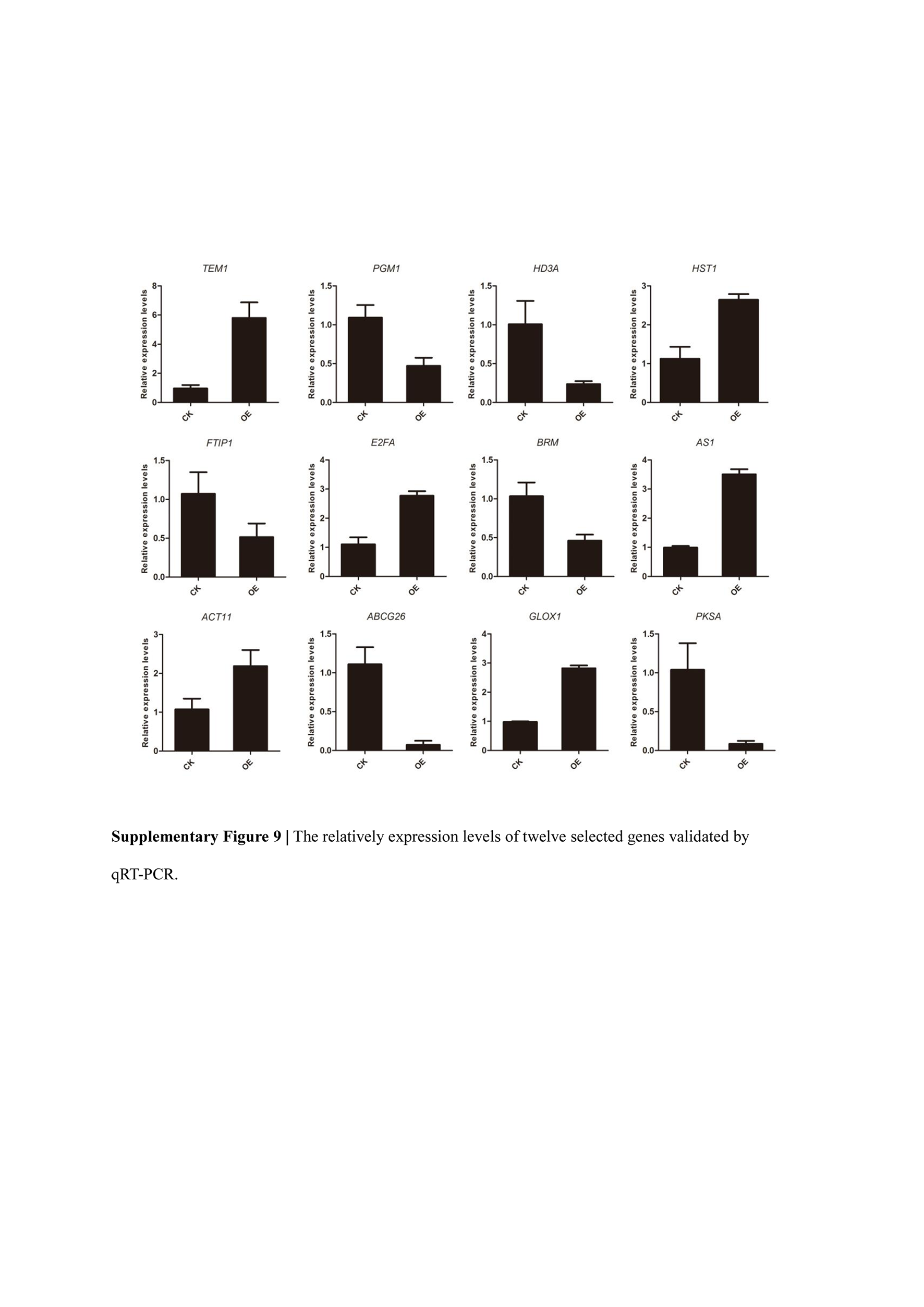

Supplement: Supplementary file 9 [file Image_9.TIF]
